# Three-Dimensional Printing in Neurosurgery: A Review of Current Indications and Applications and a Basic Methodology for Creating a Three-Dimensional Printed Model for the Neurosurgical Practice

**DOI:** 10.7759/cureus.33153

**Published:** 2022-12-30

**Authors:** Donika Vezirska, Milko Milev, Lili Laleva, Vladimir Nakov, Toma Spiriev

**Affiliations:** 1 Department of Neurosurgery, Acibadem City Clinic University Hospital Tokuda, Sofia, BGR

**Keywords:** medical education, patient-specific surgical instruments, patient-specific implants, surgical simulation, preoperative planning, 3d printing

## Abstract

Introduction

Three-dimensional (3D) printing is an affordable aid that is useful in neurosurgery. It allows for better visualization and tactile appreciation of the individual anatomy and regions of interest and therefore potentially lowers the risk of complications. There are various applications of this technology in the field of neurosurgery.

Materials and methods

In this paper, we present a basic methodology for the creation of a 3D printed model using only open-source software for medical image editing, model generation, pre-printing preparation, and analysis of the literature concerning the practical use of this methodology.

Results

The literature review on the current applications of 3D printed models in neurosurgery shows that they are mostly used for preoperative planning, surgical training, and simulation, closely followed by their use in patient-specific implants and instrumentation and medical education. Materialise^TM^ Mimics is the most frequently used commercial software for a 3D modeling for preoperative planning and surgical simulation, while the most popular open-source software for the same applications is 3D Slicer. In this paper, we present the algorithm that we employ for 3D printing using Horos^TM^, Blender, and Cura software packages which are all free and open-source.

Conclusion

Three-dimensional printing is becoming widely available and of significance to neurosurgical practice. Currently, there are various applications of this technology that are less demanding in terms of technical knowledge and required fluency in medical imaging software. These predispositions open the field for further research on the possible use of 3D printing in neurosurgery.

## Introduction

The field of neurosurgery has always required an excellent three-dimensional (3D) perspective. Magnetic resonance imaging (MRI) and computed tomography (CT) images have improved the preoperative planning and the visualization of spatial relations between normal anatomical structures and pathological objects. However, their main disadvantage is the lack of an additional dimension that shows finer details and correlations of the object of interest and may result in amplified errors in the planning of procedures involving critical anatomical structures [1]. Such a problem has motivated the need for 3D reconstructions of preoperative radiological data that have to be visualized in the most understandable and useful way, adjusted to the level of expertise of the surgeon and the goal of the operation. There are different types of virtual spatial visualization used for preoperative planning, including 3D software reconstructions with neuronavigation systems [2], commercial software for preoperative planning [3], open-source packages that can be adapted for preoperative planning [4,5], and physical 3D printed models derived from CT or MRI data [6,7].

While 3D reconstructions that appear on a monitor can be rotated in different ways and seen from many perspectives, they still reside on a flat 2D screen. An attempt to better represent 3D anatomical models and clinical images has been made with the development of holographic screens [8] or portable augmented reality systems [9]. Nevertheless, these techniques do not offer the same tactile experience that physical models do. Currently, the creation of physical 3D reconstructed objects is possible with the 3D printing technology that has become much more affordable [10] and approachable for a wider audience. Currently, the application of 3D printing technology is used to plan incisions [11], craniotomies [12], approaches [10], bony reconstructions [13], and pedicle screw insertions [14,15]. Another field for the development of this method is in the anatomical teaching at the university curriculum and in the expansion of accessibility of surgical simulations to trainees and neurosurgeons from all levels of expertise [3,11]. There have also been various reports of 3D printed spinal instrumentation guides and personalized 3D printed titanium cages for spinal surgery [16-18], whereas cranial surgery has benefitted greatly from custom-made 3D printed synthetic implants for cranioplasty [12].

Three-dimensional printing is mostly based on the concept of additive manufacturing which produces a layered 3D object from 2D images. The 3D models can be generated by computer-aided design (CAD) or a digital camera and photogrammetric software. CAD models are stored as an STL file and the slicer software converts it into a series of layers which then is being exported as G-code for instructions to the specific 3D printer. There are several types of printing processes that are suitable for 3D models for neurosurgical applications - stereolithography, fused deposition modeling, multi-jet modeling, etc. [11,12]. Different materials such as silicone, sponge, and rubber fiber can be used to supplement the 3D printed models in order to imitate delicate and soft tissue structures [6,11].

At the current state of neurosurgical practice, 3D printing is not routine in most departments, due to the technical aspect of the process and the specific knowledge needed for 3D modeling and segmentation of radiological data, requiring specialized software. Moreover, there are few descriptions of the formal steps in the process of creation of a 3D printed model for neurosurgical preoperative planning and training [19].

This study aims to review the current indications and applications of 3D printing technology in the field of neurosurgery, as well as to provide a basic methodology for creating a 3D printed model from radiological examinations for the goals of the neurosurgical practice.

## Materials and methods

A review of the literature was performed to assess the current use of 3D printing in the field of neurosurgery. Two medical databases (Google Scholar and PubMed) were searched. Inclusion criteria included articles referencing both “preoperative planning/neurosurgery” and "3d printing/neurosurgery" in addition to common variations of these terms. The results were then manually filtered according to more specific criteria; only human studies related to the skull, brain, or spine were considered. Only studies published in English were included.

In the illustrative description of the process of printing 3D neurosurgical models, we have utilized three main software packages-Horos^TM^, Blender software, and Cura. Horos^TM^ (https://horosproject.org) is an open-source imaging software with 3D volumetric rendering and 3D modeling features, as well as the ability to export the 3D model in an STL file, suitable for 3D print preparation. Blender software (https://www.blender.org) is an open-source 3D computer graphics toolset used in the creation of animated films, visual effects, 3D printed models, etc. Cura (Ultimaker, ultimaker.com) is an open-source software used as a slicing application to transfer STL file data to GCODE data, which is the hardware-level file format for 3D printing.

All three software packages are open source and free to download from the respective sites.

## Results

The main applications we outline here are preoperative planning, surgical simulation, patient-specific surgical implants, patient-specific surgical instruments, and medical education. Patient education is also a widespread application for 3D printed models [3] but it will not be the object of the current article as it should be discussed in another aspect (Table 1).

**Table 1:** A review of the current applications of 3D printing in neurosurgery and the specifics of the models that were produced AVM, arteriovenous malformation; 3D, three-dimensional. Preoperative planning: Refs. [3,6,10,12,13,15,18-32]. Surgical training and simulation: Refs. [3,6,10,11,13-20,22,23,26,27,29-32]. Patient-specific surgical implants: Refs. [12,13,16-18]. Patient-specific surgical devices: Refs. [12,14,16-18,26,28,33]. Medical education: Refs. [19,32,34].

Application	Indications	Elements of the models
Preoperative planning	Surgery of aneurysms and brain AVM, pituitary tumors, low-grade gliomas, vertebral tumor resections, pedicle screw insertions, endoscopic third ventriculostomy, craniosynostosis	Cerebral aneurysms, vessel networks, skull base, tumors, anatomical features of hydrocephalus, skulls
Surgical training and simulation	Skull base surgery, neuroendoscopy, aneurysm clipping, endovascular procedures, external ventricular drainage, cervical and thoracic spinal fixations	Cerebral aneurysms, skull base, tumors, specific regional neurovascular structures
Patient-specific surgical implants	Extensive resection of vertebral tumors where individualized implant is needed; tumors with extension to the spine, occipitocervical fixation, lumbar fixation	Custom-made rods, locking screws, vertebral replacement, anterior fusion cage, a titanium patient-specific implant with a solid occipital plate, two solid cervical anchors and a porous occiput-C2 strut, customized cranial bone flaps
Patient-specific surgical devices	Extensive resection of vertebral tumors and tumors with extension to the spine, occipitocervical fixation	Patient-specific jigs for bone resection and following reconstruction, navigation molds, headrests for frameless gamma knife surgery and guides for Le Fort osteotomies, patient-specific attachment for deep brain stimulation microelectrode placement
Medical education	Anatomy teaching	Brain, skull, and spine

We briefly reviewed the software used for each of the applications. Materialise Mimics [3,11,20] (Materialise Mimics, Belgium) is the most popular commercial software for a 3D modeling for preoperative planning and surgical simulation, while the most frequently used open-source software for the same applications is 3D Slicer™ (https://www.slicer.org/) [15,21-23]. Three-dimensional modeling for white matter tracts is possible with StealthViz™ (Medtronic Inc., Dublin, Ireland) and nordicBrainEx (Nordic Neuro Lab, Bergen, Norway) [24]. PrusaSlicer™ [21,25] (https://www.prusa3d.com/) and Ultimaker Cura [15] (https://ultimaker.com/) are mentioned as the most used open-source slicer softwares, needed to create 3D printable files from stereolithographic file (STL file), which is the file format exported from the medical imaging software. Publications about patient-specific implants (PSIs) and surgical instruments mention computer-aided design/ computer-aided manufacturing (CAD/CAM) commercial software like Anatomics C3D [18] (Anatomics, Melbourne, Australia), Amira [19] (Thermo Fisher Scientific, Waltham, MA, USA) and Autodesk Netfabb [19] and Autodesk 3ds Max [12] (Autodesk, San Francisco, CA, USA).

In this paper, we aim to describe a method that we use to create a 3D printable model from the patient’s DICOM CT or MRI data.

The method for 3D modeling using OsiriX (Pixmeo Bernex, Switzerland), now applicable to Horos software, which is free and open source, has been previously described in detail elsewhere [7]. In short, using the 3D modeling window the basic 3D model is tailored using the “Sculpt” tool with the three options available: the “backspace” key that cuts inside the outlined area, the “return” key that cuts outside the outlined area, and the “tab” key that reconstructs the pixels inside the outlined area. 

**Figure 1:**
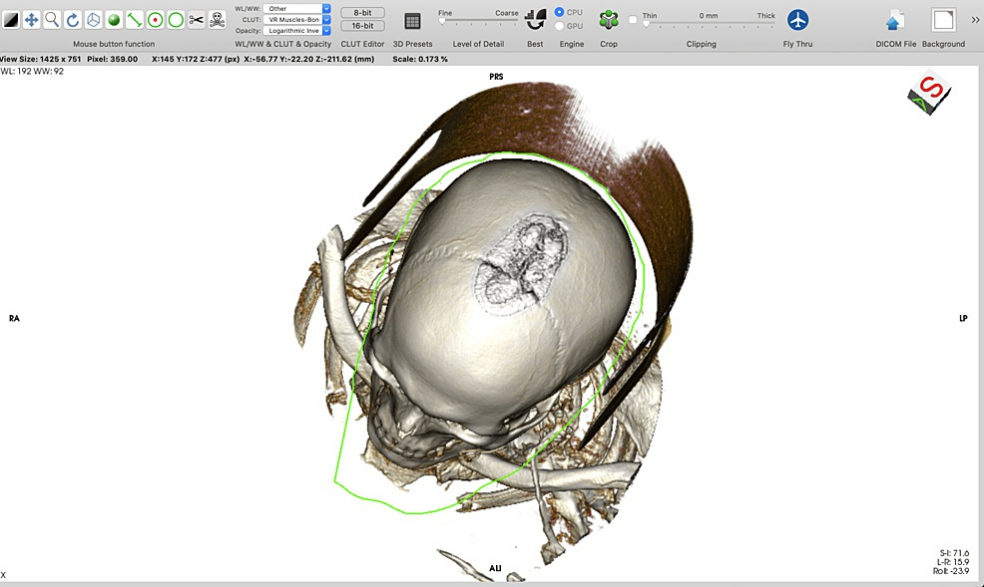
Basic 3D modeling in Horos™ software (https://horosproject.org) using the “Sculpt” tool. Cut outside the outlined area using the “Return” key. 3D, three-dimensional.

**Figure 2:**
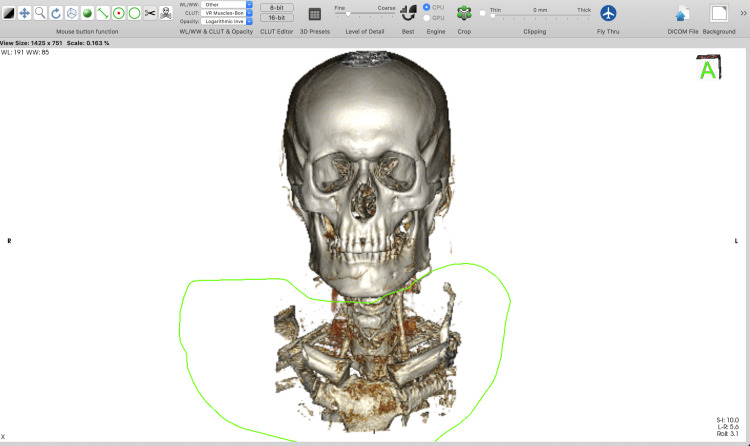
Cut inside the outlined area using the “Backspace” key.

**Figure 3:**
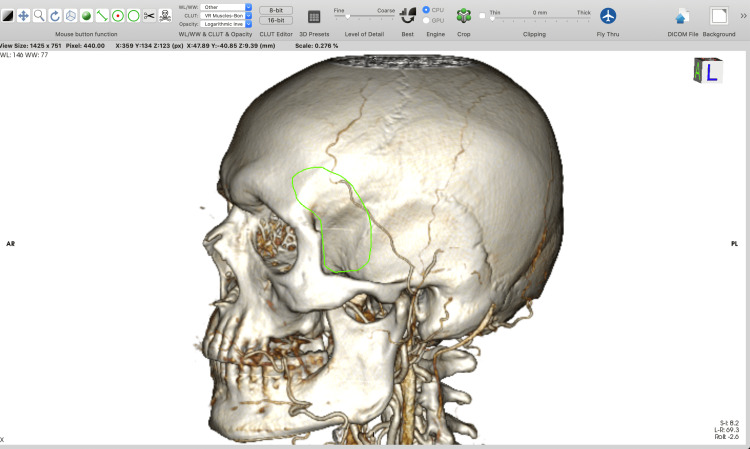
Example of tailoring a minipterional craniotomy using the “Backspace” key to remove all the voxels inside the outlined area.

**Figure 4:**
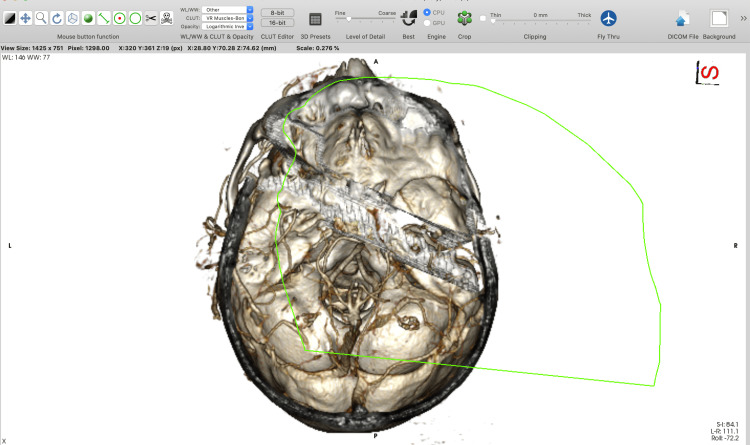
Reconstruction of the voxels inside the region of interest by using the “Tab” key.

**Figure 5:**
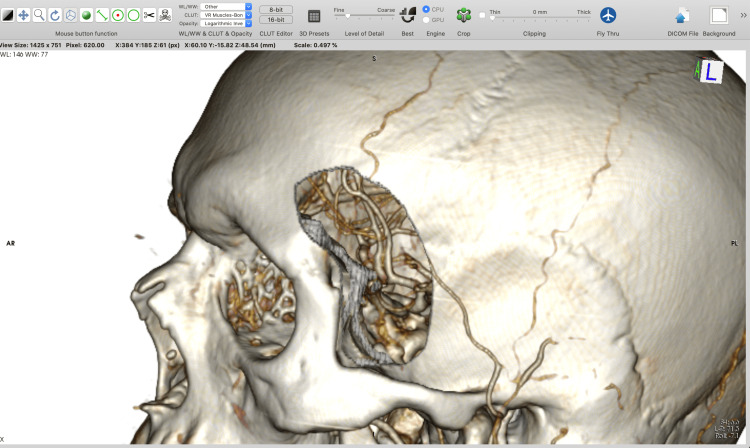
A final lateral view of the minipterional craniotomy simulation.

**Figure 6:**
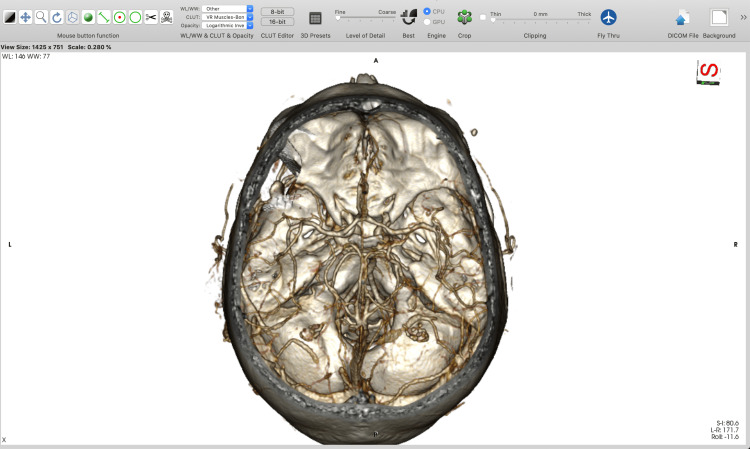
A final superior view of the minipterional craniotomy simulation.

Using the “backspace” key, which cuts inside the outlined area, allows the removal of all unnecessary parts of the 3D model. Using the “return” key, which cuts outside the outlined area, allows the selection of only the designated anatomical region of interest. The option with the “tab” key is used when a specific surgical approach (e.g. pterional) has to be tailored. The "backspace" key removes all the voxels inside the outlined area. However, voxels inside the region of interest that were cut must be reconstructed (by using the “Sculpt” tool along with the “tab key”) to simulate only a bone-opening window and to visualize the content in the cranium (Figures 1-6).

**Figure 7:**
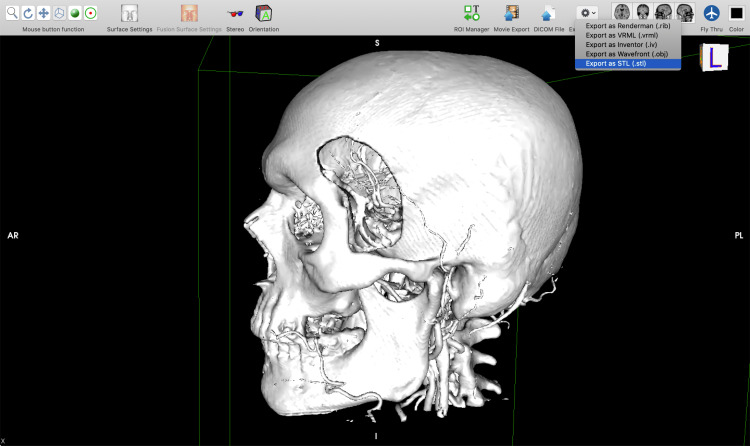
Three-dimensional surface rendering window and exporting an STL file in HorosTM (https://horosproject.org) software. STL, stereolithographic.

After that, the 3D surface rendering window is opened where an STL model is generated (Figure 7).

**Figure 8:**
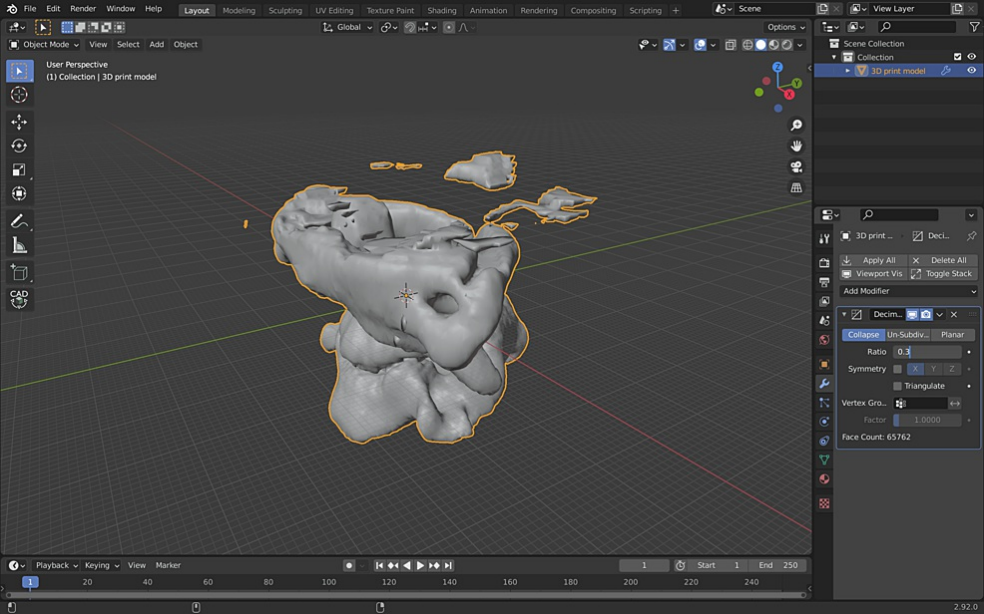
Importing and decimating the primary 3D model in Blender (Blender, blender.org). 3D, three-dimensional.

The STL file is then imported to Blender software, where first the mesh (the structural build of a 3D model consisting of polygons) of the 3D model is decimated using the Decimate modifier which will drastically decrease the mesh complexity and will allow for easier 3D modeling (Figure 8).

**Figure 9:**
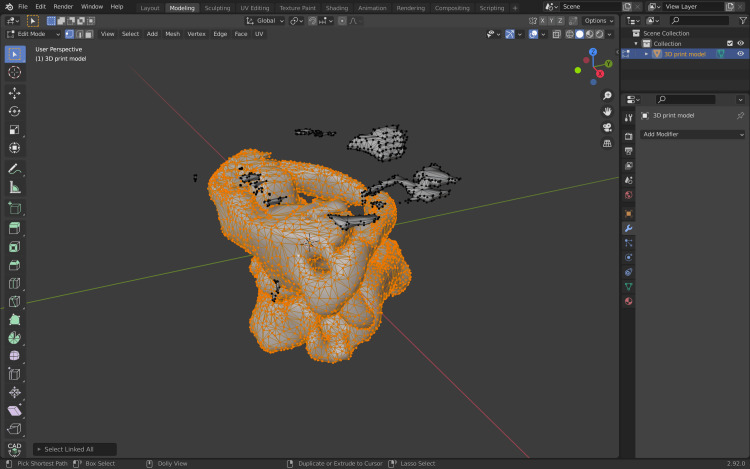
Selection of all linked vertices of the 3D model. 3D, three-dimensional.

The next step will allow for cleaning the 3D model of unwanted vertices. Select one vertex from the 3D model and press Command +L (Ctr+L) which will allow for the selection of any linked vertices of the 3D model (Figure 9).

**Figure 10:**
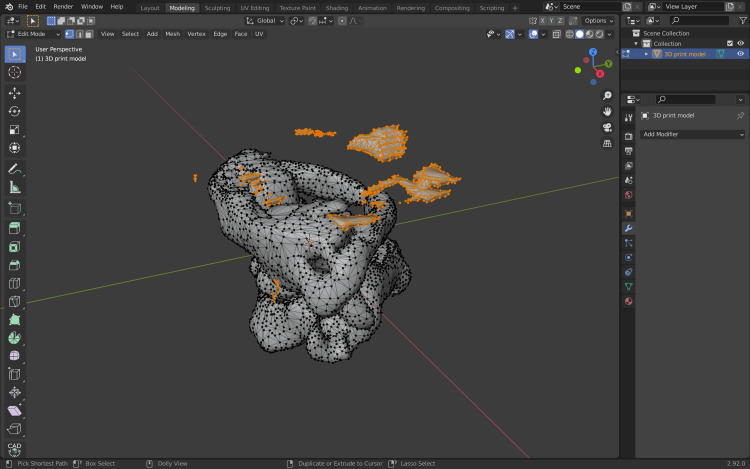
Inversion of the selection and selecting any vertex that is not linked to the models.

Then, Command +I (Ctrl+L) is pressed; this will invert the selection and select any vertex that is not linked to the models (Figure 10).

**Figure 11:**
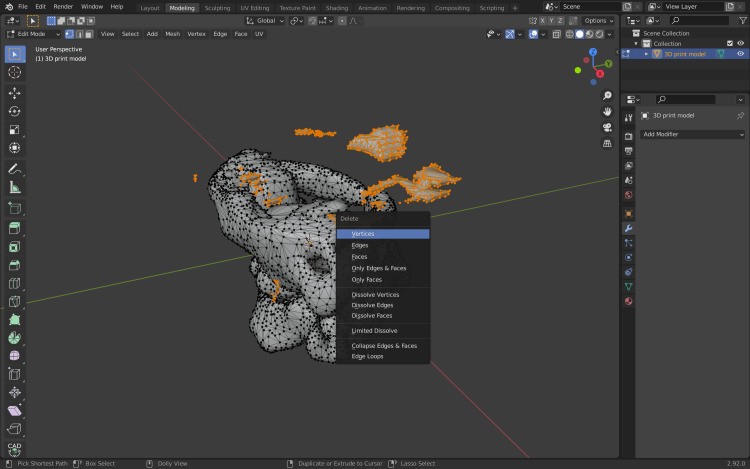
Deleting the vertices that are non-linked to the model.

Then, the key “X” is pressed; this will delete the non-linked to the model vertices (Figure 11).

**Figure 12:**
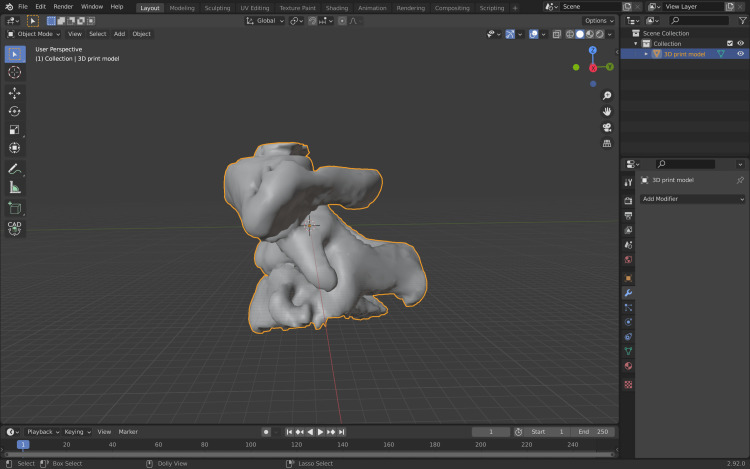
Final 3D model before being exported to Cura software (Ultimaker, ultimaker.com). 3D, three-dimensional.

Irregularities can be corrected in the Sculpt module of the software. After these steps, the 3D model is ready to be exported from Blender software as an STL file from File/export/export as STL file (Figure 12).

**Figure 13:**
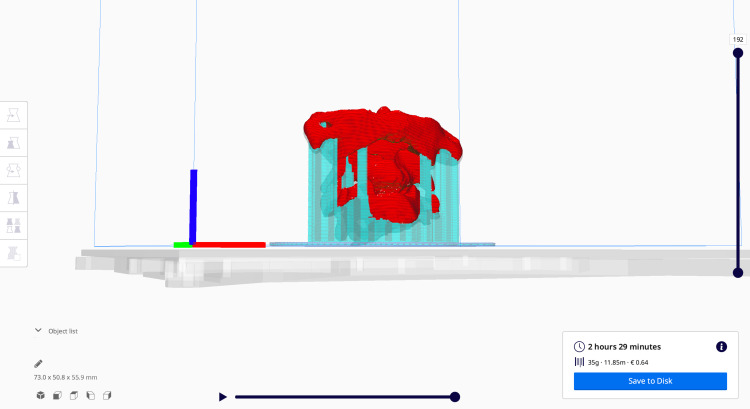
3D model in Cura software (Ultimaker, ultimaker.com) sliced before being exported to G-code file for 3D printing. 3D, three-dimensional.

The latter is imported in Cura software (Ultimaker, ultimaker.com), which is an open-source slicing application for 3D printers where the exact position and rotation of the 3D model is selected on the printing bed (Figure 13).

Then we use the following parameters for 3D printing using PLA (polylactic acid) filament (Table 2).

**Table 2:** Basic parameters for 3D printing, with Cura software (Ultimaker, ultimaker.com) 3D, three-dimensional.

Parameter	Setting
Layer height	0.2 mm (standard quality)
Build plate adhesion	Brim
Generate support (true)	Everywhere
Infill density	15-20%
Printing speed	Between 60 and 80 mm/sec
Printing temperature	215 degrees
Bed temperature	60 degrees

We recommend leveling the printer before each 3D print.

**Figure 14:**
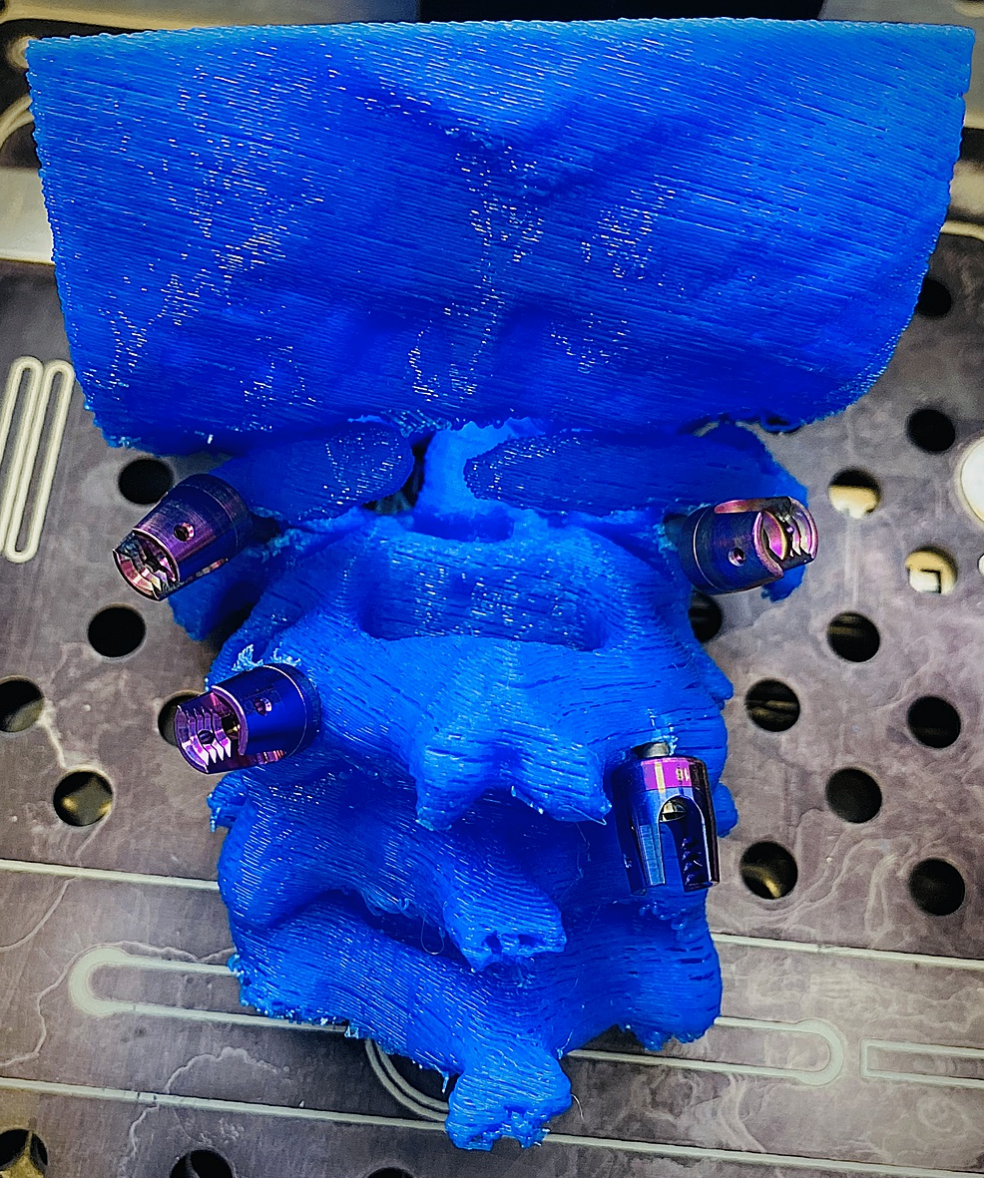
Simulation of preoperative screw placement on a 3D printed model of C1-C2 subluxation and C2 dens fracture case: posterior view. 3D, three-dimensional.

**Figure 15:**
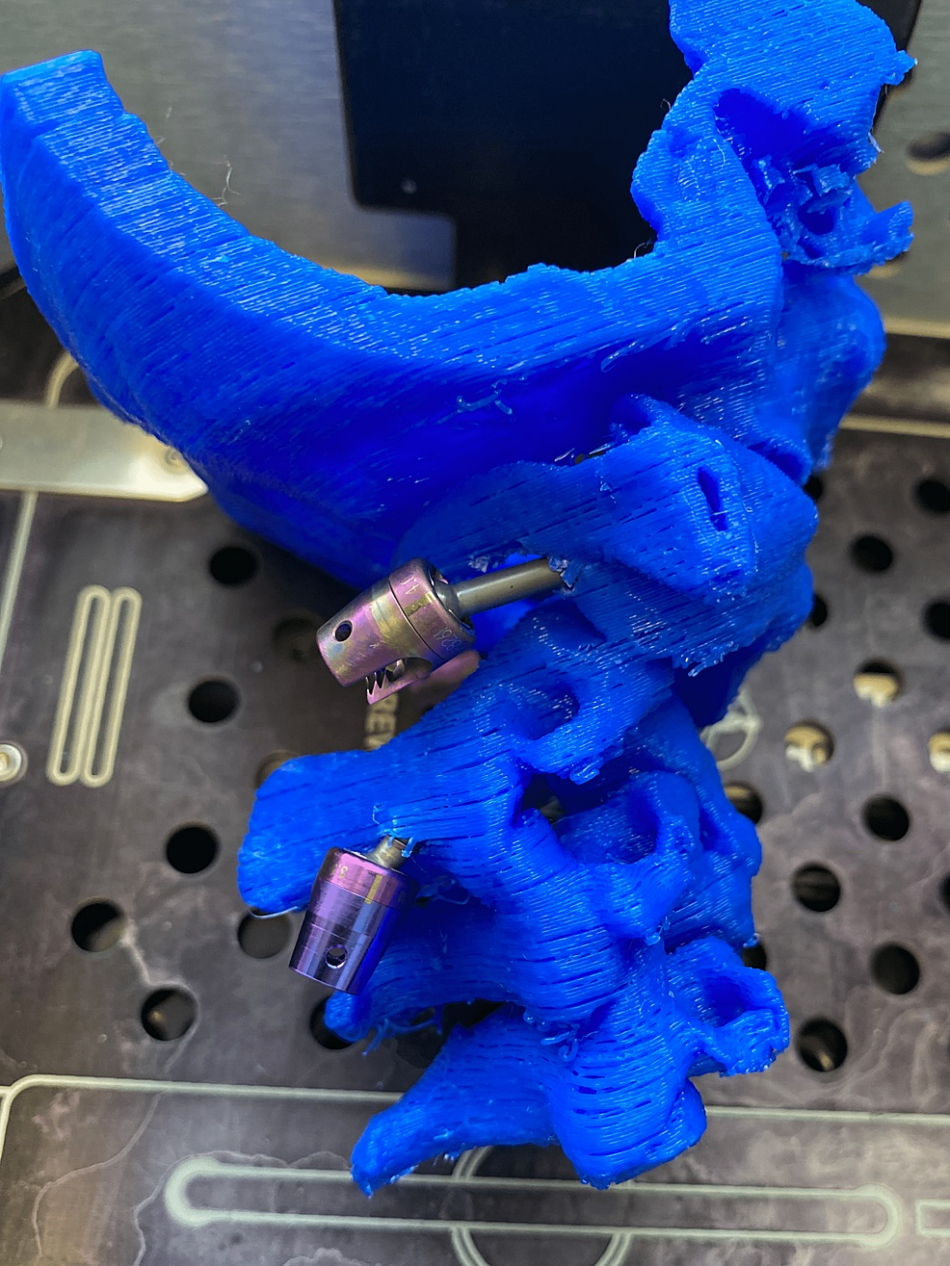
Simulation of preoperative screw placement on a 3D printed model of C1-C2 subluxation and C2 dens fracture case: lateral view. 3D, three-dimensional.

The final print is used depending on the specific case needs. In Figure 5, we present a 3D printed model utilized for preoperative planning of a complex case of C1-C2 subluxation and C2 dens fracture in a patient with Down syndrome who is a professional athlete. We first performed a C1 anterior screw fixation and a following C1-C2 posterior fixation. The posterior C1-C2 fusion was preoperatively trained on the 3D printed model. The postoperative course is uneventful during the one-year follow-up and the patient continued his active sports career (Figures 14, 15).

## Discussion

Neurosurgery is a field that requires both tridimensional spatial awareness of the region of interest. Three-dimensional printing is an excellent aid to the surgeon in this regard, while it has also proven to be accurate in the representation of finer details in medical imaging. According to Thawani et al., the 3D printed model reproduces the imaging findings by 0.1 mm [10]. This makes the technique useful for various applications in the field of neurosurgery.

Five major applications in neurosurgery can be outlined for 3D printing - preoperative planning, simulation training, patient-specific surgical instruments, PSIs, and medical education.

Preoperative planning

Three-dimensional printing has been used to plan approaches and visualize the normal anatomy and the pathological regions for both spinal [14,15,17,18,23,26] and cranial procedures [21,24,25,27-29].

Spinal Procedures

The literature review shows that cervical, cervicothoracic, thoracic, thoracolumbar, and lumbar procedures such as pedicle screw insertions and various fixations can be planned with preprinted 3D models [14,15,23]. Three-dimensional printing has proved to be useful in planning the stabilization of extremely difficult cases of spinal instability [18,23]. It is a method that could potentially aid the training for cervical fixation [14,17,18] while simultaneously improving patient safety for a technically difficult procedure. Rare cases of difficult-to-plan wide vertebral tumor resections [23] can be facilitated by a PSI tailored to every patient’s anatomy. The use of such implants reduces the risk of misalignment, failure of instrumentation, and worsened vertebral column support [16]. This can also reduce the costs for departments resulting from the use of instrumentation sets from different companies - a single company or even a 3D printing laboratory within the hospital can be used to produce the needed materials. Govsa et al. [14] present a case study for the use of 3D printing for exploring the fracture locations, pedicle sizes, and positions of the vertebral artery in fixation of C1 fractures, while Thayaparan et al. [18] also describe an operative case of C1 fracture in which they used a 3D printed model to plan the neutral position for fixation, skin incision, and muscle dissection, and the desired screw sizes, trajectories, and entry points to secure the single-piece PSI to the occiput and C2-C4. Mobbs et al. [17] report the use of a 3D printed model to plan the resection margins for a C1-C2 osteolytic chordoma, followed by occipitocervical fixation and a patient-specific titanium cage implant in the place of the resected C1-C2 vertebrae. These studies show that 3D printing is suitable for planning complicated cases where routine approaches and implants are not sufficient. Resection of vertebral tumors is frequently associated with postoperative spinal instability while fitting a pre-made implant in the resected region is oftentimes impossible and of no use because of the uneven endplates and different spine curvature angles in different zones of the cavity.

Different authors [15,23] have described the use of 3D printed models for planning pedicle screw insertions for upper and middle thoracic vertebral fixation and spinal deformities. There are also several cases of complex spinal column tumors (cervical, thoracic, and lumbosacral) in which a 3D printed model of the lesion and the surrounding anatomy was made and referenced intraoperatively for the osteotomy borders [17,23]. Some of the models had the option to be disassembled, which allowed for step-by-step visualization of the surgical procedure and familiarization with different regions of the tumor infiltration. Statistically significant superiority of the results achieved by preoperative planning with 3D printed personalized spine models compared to both the freehand and the intraoperative neuronavigation techniques were reported but multicenter research needs to be done, especially on the comparison with the neuronavigation [15]. However, available research shows that preoperative planning by means of 3D printed personalized spine models has improved the accuracy of screw placement - they can be used in more complex screw insertions that are near major neural and vascular structures [14].

Cranial Surgery

Three-dimensional printing has an even wider application in the planning of cranial procedures - the literature review shows the utilization of mostly tumor [11], aneurysm [25,29], and brain arteriovenous malformation (AVM) [3] models. Currently, there is also an option for preoperative planning in both vascular neurosurgery and endovascular interventions [28]. Other possibilities also involve a large number of regions of interest including intricate anatomic details such as cranial nerves [27] and nerve fiber tracts [24]. The aforementioned can be 3D printed with acceptable accuracy for the need of complex cranial cases [21].

Gargiulo et al. [24] describe a case of a low-grade glioma where models of nerve fiber tracts derived from fiber tracking were 3D printed. A comparison was made between two software platforms (nordicBrainEx and StealthViz) in accordance with the anatomic accuracy of projections of fibers of the corpus callosum, arcuate fasciculus, and right and left motor and sensory tracts and optic tracts. This revealed nordicBrainEx to have better overall performance. The challenge of accurate integration of information from CT, diffusion tensor imaging (DTI), and MRI still remains but a 3D printed model with such integration by nordicBrainEx was successfully used for preoperative planning. Gillett et al. [21] depict the making of colored 3D printed models for pituitary tumors and vessels and structures adjacent to the pituitary gland and the tumor within approximately 30 mm. This was possible because of the use of multiple 3D printing technologies - PBF (powder bed fusion), MJ (material jetting), VP (vat photopolymerization), and MEX (material extrusion). As MJ and PBF need commercial printing, this can increase the price of the model and make the technique less accessible to the departments that may need them. Lin et al. [27] show the application of 3D printed models in three cases of skull base surgery with the inclusion of cranial nerves that are in relation to the tumors (acoustic neuroma and sellar region tumors). The approaches can also be simulated from craniotomy to tumor extirpation. Ganguli et al. [11] describe their application for optimal patient positioning, planning of tailored approaches for skull base surgery, and resections of lesions near the motor cortex. Overall, the studies show that 3D printed models are very useful for the general planning of approaches and tumor resections but the cases where white matter tracts are involved are still very difficult to model. The integration of data from different imaging modalities is a lingering issue that is yet to be resolved.

Aneurysm surgery has long been one of the most difficult areas in modern neurosurgery. With the help of current 3D printing technology, preoperative 3D models can facilitate preoperative planning and potentially increase patient safety. Błaszczyk et al. [25] present the making of 3D printed models of aneurysms and their parent vessels in the complex of the circle of Willis. However, their models were not hollow and they were much more rigid than the real anatomic elements, seeing as they used PLA to produce them - clipping simulation was not possible. Malilay et al. [29] mention the use of 3D printed models for better visualization and spatial orientation on aneurysms’ location and position from different points of view and for the optimal choice of aneurysm clips; planning an incision and a craniotomy according to a specific tumor or aneurysm localization is also possible. Other authors [3,28] report the use of 3D printing in brain AVM cases to evaluate the nidus with the relevant feeding artery and draining veins, the adjacent region, and the potential bleeding area and to optimize the approach, whether it be endovascular or neurosurgical. While this is certainly a great preoperative planning tool for all who practice vascular neurosurgery, one should note that there was no substantial proof of exact anatomic correspondence to the real anatomy of the AVM, while the aneurysm structure can be replicated with acceptable accuracy and with relatively low-cost materials.

Surgical training and simulation

Three-dimensional printed models and simulators have a growing role in the training of residents and young neurosurgeons for both simpler procedures such as external ventricular drainage [30] and pedicle screw insertions [15] and more complex ones such as aneurysm clipping [6] and neuroendoscopy surgical scenarios [22].

Spinal Procedures

Three-dimensional printing for surgical simulation in spinal procedures is mostly used for pedicle screw placement [12,15], especially in technically difficult cervical and upper thoracic screw fixation. Cornejo et al. [12] describe the use of 3D printed models to simulate different pedicle screw insertions, including C2 laminar screw placements. This can aid in the familiarization with the anatomy and the haptic feedback one will go through in a surgery, especially for an inexperienced surgeon who does not perform such operations routinely. Kaya et al. [15] mention using 3D printed models for preoperative simulation and familiarization with the specific anatomy in upper thoracic pedicle screw fixation cases. Another option to increase the accuracy of the pedicle screw insertions is by spinal neuronavigation. However, neuronavigated pedicle screw insertion relies on the lack of position changes of the reference landmark and spinal stability, which is rarely the case with fractured thoracic vertebrae. The authors used 3D printed models and concluded that the preoperative simulation of the pedicle screw insertions has the potential to reduce violations to the spinal canal and vascular injuries, seeing that the results from a series of 174 cases show statistically significant better accuracy of the pedicle screw placement with 3D printed preoperative guidance (96.6%) than without it (83.6%).

Cranial Procedures

Mostly, the applications of 3D printing are for simulations of cranial procedures for aneurysm clipping as well as placement of external ventricular drainage [30], neuroendoscopic techniques [12,22,31], and a relatively accurate experience of a full cranial approach from the bone flap to the lesion [20]. Cornejo et al. [12] review the use of 3D printed models for the simulation of neuroendoscopic techniques (third ventriculostomy, pineal biopsy), different cranial approaches, external ventriculostomy and craniosynostosis as well as different scenarios for complications such as intraoperative venous sinus injury. Hsieh et al. [22] present the use of 3D printed models of the skull base and sinuses to simulate steps from modified endoscopic Lothrop, endoscopic anterior craniofacial resection, and transpterygoid and transclival approaches - haptic feedback of the instruments was also positively assessed by the participants. Multiple authors have described the preparation of multicolor 3D printed models [19] that were capable of simulation of a full surgical approach and tumor resection for different lesions with accurate inclusion of all adjacent structures [20]. This approach to preoperative planning could optimize the width of the craniotomy while aiding the preservation of deep structures but it is still difficult to achieve adequate haptic feedback and the time and effort needed for the construction of an informative 3D printed model may be nonreciprocal to the benefits that are expected from it. McGuire et al. [10] present a comprehensive review of the use of 3D printed models in both vascular neurosurgery and endovascular interventions including the simulation of AVM embolization and aneurysm coiling. Weinstock et al. [31] present the use of 3D printed models for the simulation of endoscopic third ventriculostomy with reportedly accurate haptic feedback. Yi et al. [30] show the application of a patient-specific 3D printed model for external ventricular drainage procedure training - an assisting tool was also 3D printed to aid in the perpendicular insertion of the catheter. This could be a good initial training for residents but it has not been proven to be more successful in the planning of trajectories than neuronavigation, so it cannot be considered reliable in a clinical setting.

Patient-specific implants

PSIs have a growing use in both spinal and cranial procedures. The literature review shows that most of the spinal uses include patient-specific cages, plates, and rods [16-18], while most of the cranial uses include customized patient-specific bone flap substitutes and bony craniofacial reconstructions [12,13]. An issue that is worth noting is the certification of the materials and machines for use in patients - this can be a significant barrier to the wider application of the technology.

Spinal

Thayaparan et al. [18] describe a case of C1 fracture in which a titanium PSI with a solid occipital plate, two solid cervical anchors, and a porous occiput-C2 strut were 3D printed. Lador et al. [16] report the use of titanium patient-specific vertebral replacement implants, as well as pre-bent carbon-fiber rods that were made in the shape of 3D printed templates. The model was created with GrabCAD (GrabCAD, Inc., Cambridge, MA, USA) and 3D printed by 4WEB Medical (4WEB Medical, Frisco, TX, USA) - this could potentially be a problem with the reproduction of the results because many institutions do not have the access to such expensive services and implants. Mobbs et al. [17] present the use of patient-specific vertebral replacement implants and a titanium anterior fusion cage after vertebral tumor resections. However, the software and the 3D printers that one needs in order to print the implants are industrial and certain licenses are required in order to produce them. Furthermore, there could be legal and administrative issues when one plans to insert these types of custom-made implants - oftentimes they are not validated by the specific insurance agency and public health administration.

Cranial

PSIs for cranial application are most frequently synthetic patient-specific cranioplasties [12] for bony defect coverage and artificial custom-made implants for orbital floor fracture reconstructions [13]. This is a method that is worth exploring in the context of postoperative enophthalmos after wider orbital extensions of the FTOZ approach and approaches that use more extensive drilling of bony structures resulting in worsened cosmesis or quality of life for the patient.

Patient-specific surgical instruments

One can observe a growing trend for the production of patient-specific 3D printed surgical instruments as well. This has been made possible by the CAD/CAM technology that is a must for the production of such objects - patient-specific jigs for protection of the dural sac during surgical resection of the vertebrae [16], tools for optimal needle insertion in cryoablation and protection of adjacent vascular structures during the procedure, 3D printed patient-specific jigs for bone resection of skull tumors and following reconstruction [26]. The latter can provide the opportunity to excise and repair the bony defect in one single operation by outlining precise boundaries for the cutting drill and designing a 3D printed flap that is a perfect fit for them. It is worth noting that the studies are based on a few cases that had to deal with very rare tumors, so the technology is not routinely used in the practice.

An aspect that can be looked into is the use of patient-specific osteotomy guides for different cranial vault deformities. Some authors suggest the use of 3D printed models as osteotomy guides for Le Fort osteotomy [12] and osteotomy guides for calvarial vault reconstruction [28]. Some of the issues that may arise with this is the cost of the CAD/CAM 3D printed model that can present an obstacle for the parents of the child, depending on the healthcare system organization in the specific country and the availability of the technology in the specific region.

Functional neurosurgery is a field where 3D printing can be of great aid - precise entry points and trajectory planning are difficult to do free hand and require an experienced functional surgeon. Therefore, another application is the creation of a 3D printed jig for deep brain stimulation surgery [33]. The jig is intended to be used as a patient-specific drill guide that is fixed to the stereotactic frame. This allows for a more precise burr hole drilling to decrease the trajectory misalignment.

Medical education

Three-dimensional printing is also becoming a great part of medical education as well. Langridge et al. [32] found 3D printed models to be superior for learning and understanding to virtual 3D imaging and all 2D educational models - there was a statistically significant difference between the results from the anatomy quizzes of trainees who used 3D printed models and those who used 2D or 3D imaging to prepare [34]. The use of multicolor 3D printed objects [19] is an affordable and effective method of improving medical education by better understanding complex relations between different objects in a region of interest. What is more, 3D printed models can be a good educational tool in countries with strict laws regarding the use of cadavers in medical school and training courses [35]. Companies like UpSurgeon even have mobile applications to go with their 3D printed simulators and offer a virtual step-by-step planning of craniotomies. Haptic feedback has been reported to be very accurate to the real anatomic details and that helps trainees with handling surgical tools in a real surgical setting [6].

Limitations of 3D printing

A potential drawback to the use of 3D printing is the time that is needed to make a model - it is still a relatively slow process that includes specific knowledge and experience in 3D modeling or CAD software that is not easy to achieve in a short period of time as well as time that cannot be spared in acute situations. Emergency surgeries are hard to plan with a 3D printed model because the technology does not allow for fast enough printing in most cases - Błaszczyk et al. [25] report their time from computed tomography angiography (CTA) acquisition to a ready model to be around 4 hours. More detailed 3D models that could be used for dissection and clipping simulation are more expensive and take longer to make [6].

Another problem that can arise from the single use of personalized 3D printed objects is the waste this would generate [35]. Options for recycling should be looked into and incorporated into the standard practice when creating models - a closed cycle would be the optimal way to both preserve the environment and reuse the material while being economical with the 3D printing filaments in the long run in order to make the process sustainable and cost-effective. A possibly different perspective to the question is that the use of patient-specific models indirectly reduces waste, e.g. single-use consumables, by increasing the efficiency and safety of the surgical procedures.

## Conclusions

Three-dimensional printing has a wide field of applications in neurosurgery including preoperative planning, models for training purposes, and PSIs and instruments. It allows for better visualization and appreciation of the individual anatomy and regions of interest and lowers the intraoperative risks for the patient.

The basic methodology for creating a 3D printed model that we outline in this paper has the potential to lay the foundation for professionals who would like to become familiar with 3D printing and its many applications, but do not have time to learn complex CAD or 3D modeling software. The paper provides an opportunity to get an initial grasp of the process and start introducing it in one’s own practice, while it presents the current indications and applications of this technology in neurosurgery.

## References

[1] Kin T, Nakatomi H, Shojima M (2012). A new strategic neurosurgical planning tool for brainstem cavernous malformations using interactive computer graphics with multimodal fusion images. J Neurosurg.

[2] Mert A, Buehler K, Sutherland GR (2012). Brain tumor surgery with 3-dimensional surface navigation. Neurosurgery.

[3] Kockro RA, Killeen T, Ayyad A (2016). Aneurysm surgery with preoperative three-dimensional planning in a virtual reality environment: technique and outcome analysis. World Neurosurg.

[4] Harput MV, Gonzalez-Lopez P, Türe U (2014). Three-dimensional reconstruction of the topographical cerebral surface anatomy for presurgical planning with free OsiriX Software. Neurosurgery.

[5] Shi H, Li Y, Wang Y (2022). The preoperative evaluation value of 3D-slicer program before microsurgical vascular decompression in patients with hemifacial spasm. Clin Neurol Neurosurg.

[6] Spetzger U, Etingold J, von Schilling A (2022). Training models for skull-base and vascular micro-neurosurgery. Skull Base Surgery.

[7] Spiriev T, Nakov V, Laleva L, Tzekov C (2017). OsiriX software as a preoperative planning tool in cranial neurosurgery: a step-by-step guide for neurosurgical residents. Surg Neurol Int.

[8] Ko K (1998). Superimposed holographic image-guided neurosurgery. Technical note. J Neurosurg.

[9] Haemmerli J, Davidovic A, Meling TR, Chavaz L, Schaller K, Bijlenga P (2021). Evaluation of the precision of operative augmented reality compared to standard neuronavigation using a 3D-printed skull. Neurosurg Focus.

[10] McGuire LS, Fuentes A, Alaraj A (2021). Three-dimensional modeling in training, simulation, and surgical planning in open vascular and endovascular neurosurgery: a systematic review of the literature. World Neurosurg.

[11] Ganguli A, Pagan-Diaz GJ, Grant L (2018). 3D printing for preoperative planning and surgical training: a review. Biomed Microdevices.

[12] Cornejo J, Cornejo-Aguilar JA, Vargas M (2022). Anatomical engineering and 3D printing for surgery and medical devices: international review and future exponential innovations. Biomed Res Int.

[13] Khomutinnikova NE, Durnovo EA, Vyseltseva YV, Gorbatov RO (2021). Digital technologies in the surgical treatment of post-traumatic zygomatico-orbital deformities. Sovrem Tekhnologii Med.

[14] Govsa F, Ozer MA, Biceroglu H, Karakas AB, Cagli S, Eraslan C, Alagoz AK (2018). Creation of 3-dimensional life size: patient-specific C1 fracture models for screw fixation. World Neurosurg.

[15] Kaya I, Cingöz İD, Şahin MC, Atar M, Ozyoruk S, Sayin M, Yuceer N (2021). Are 3D printing templates an advantage in upper thoracic pedicle screw fixation?. Cureus.

[16] Lador R, Regev G, Salame K, Khashan M, Lidar Z (2020). Use of 3-dimensional printing technology in complex spine surgeries. World Neurosurg.

[17] Mobbs RJ, Coughlan M, Thompson R, Sutterlin CE, Phan K (2017). The utility of 3D printing for surgical planning and patient-specific implant design for complex spinal pathologies: case report. J Neurosurg Spine.

[18] Thayaparan GK, Owbridge MG, Thompson RG, D'Urso PS (2020). Patient-specific processes for occipitocervical fixation using biomodelling and additive manufacturing. J Clin Neurosci.

[19] Kosterhon M, Neufurth M, Neulen A (2020). Multicolor 3D printing of complex intracranial tumors in neurosurgery. J Vis Exp.

[20] Mussi E, Mussa F, Santarelli C (2020). Current practice in preoperative virtual and physical simulation in neurosurgery. Bioengineering (Basel).

[21] Gillett D, Bashari W, Senanayake R (2021). Methods of 3D printing models of pituitary tumors. 3D Print Med.

[22] Hsieh TY, Cervenka B, Dedhia R, Strong EB, Steele T (2018). Assessment of a patient-specific, 3-dimensionally printed endoscopic sinus and skull base surgical model. JAMA Otolaryngol Head Neck Surg.

[23] Leary OP, Crozier J, Liu DD (2021). Three-dimensional printed anatomic modeling for surgical planning and real-time operative guidance in complex primary spinal column tumors: single-center experience and case series. World Neurosurg.

[24] Gargiulo P, Árnadóttir Í, Gíslason M, Edmunds K, Ólafsson I (2017). New directions in 3D medical modeling: 3D-printing anatomy and functions in neurosurgical planning. J Healthc Eng.

[25] Błaszczyk M, Jabbar R, Szmyd B, Radek M (2021). 3D printing of rapid, low-cost and patient-specific models of brain vasculature for use in preoperative planning in clipping of intracranial aneurysms. J Clin Med.

[26] Wu CT, Lu TC, Chan CS, Lin TC (2021). Patient-specific three-dimensional printing guide for single-stage skull bone tumor surgery: novel software workflow with manufacturing of prefabricated jigs for bone resection and reconstruction. World Neurosurg.

[27] Lin J, Zhou Z, Guan J (2018). Using three-dimensional printing to create individualized cranial nerve models for skull base tumor surgery. World Neurosurg.

[28] LoPresti M, Daniels B, Buchanan EP, Monson L, Lam S (2017). Virtual surgical planning and 3D printing in repeat calvarial vault reconstruction for craniosynostosis: technical note. J Neurosurg Pediatr.

[29] Malilay OR, Ferraris KP, Navarro JE (2021). Editorial. Neurosurgical planning in a low-resource setting using free open-source three-dimensional volume-rendering software. Neurosurg Focus.

[30] Yi Z, He B, Deng Z, Liu Y, Huang S, Hong W (2021). A virtual reality-based data analysis for optimizing freehand external ventricular drain insertion. Int J Comput Assist Radiol Surg.

[31] Weinstock P, Rehder R, Prabhu SP, Forbes PW, Roussin CJ, Cohen AR (2017). Creation of a novel simulator for minimally invasive neurosurgery: fusion of 3D printing and special effects. J Neurosurg Pediatr.

[32] Langridge B, Momin S, Coumbe B, Woin E, Griffin M, Butler P (2018). Systematic review of the use of 3-dimensional printing in surgical teaching and assessment. J Surg Educ.

[33] Ang J, Zhang JJ, Yam M, Maszczyk T, Ng WH, Wan KR (2022). Clinical application of a stereotactic frame-specific 3D-printed attachment for deep brain stimulation surgery. World Neurosurg.

[34] Ye Z, Dun A, Jiang H, Nie C, Zhao S, Wang T, Zhai J (2020). The role of 3D printed models in the teaching of human anatomy: a systematic review and meta-analysis. BMC Med Educ.

[35] Zhu C, Li T, Mohideen MM, Hu P, Gupta R, Ramakrishna S, Liu Y (2021). Realization of circular economy of 3D printed plastics: a review. Polymers (Basel).

